# A new species, first report of a male and new distributional records for three species of *Pteroptrix* (Hymenoptera: Aphelinidae) from China

**DOI:** 10.3897/BDJ.5.e12387

**Published:** 2017-03-20

**Authors:** Ye Chen, Cheng-De Li

**Affiliations:** 1 School of Forestry, Northeast Forestry University, Harbin, China

**Keywords:** *Pteroptrix pedicellata*, Chalcidoidea, Coccophaginae, taxonomy.

## Abstract

**Background:**

The Chinese fauna of *Pteroptrix* currently includes 26 species, of wihich 19 species were described originally as new from China: 2 by Howard (1907), 2 by Compere (1953), 1 by Flanders (1966), 1 by Viggiani and Ren (1986), 4 by Huang (1991), 4 by Huang et al. (1992), 1 by Viggiani and Ren (1993), 4 by Huang (1994).

**New information:**

*Pteroptrix pedicellata* **sp. n.** is described in detail and illustrated; the male of *P. processa* (Huang) is reported for the first time. New distributional data for three species, *P. leptocera* (Huang), *P. orientalis* (Silvestri) and *P. processa* (Huang), are also provided from China.

## Introduction

The genus *Pteroptrix* was established by Westwood (1833) with *Pteroptrix dimidiatus* Westwood as the type species and currently comprises 71 valid species (Noyes 2016). All the species of the genus are parasitoids of armoured scale insects (Hemiptera: Diaspididae) which are economically important pests. The taxonomy of the genus had been highly controversial for a long time until Viggiani and Garonna (1993) proposed *Archenomus* Howard and *Aphelosoma* Nikol'skaya as synonyms of *Pteroptrix*. Faunal contributions to the systematic study of the genus had been made by several authors, such as Hayat (1979) on the Australian fauna; Prinsloo and Neser (1990) on the southern Afrotropical fauna; Nikol'skaya and Yasnosh (1966), Viggiani and Garonna (1993) on the European fauna; Abd-Rabou (2002) on the Egyptian fauna; Myartseva (2009) on the Mexican fauna; Hayat (1998), Hayat and Khan (2010) on the Indian fauna; Huang (1994) on the Chinese fauna.

## Materials and methods

All specimens in the present study were collected by sweeping, or yellowpan trapping, then dissected and mounted in Canada balsam on slides following the method described by Noyes (1982). Photographs were taken with a digital CCD camera attached to an Olympus BX51 compound microscope and final modifications to the images were made using Adobe Photoshop. Most measurements were made from slide-mounted specimens using a reticle eyepiece in a microscope, except the total body length (excluding the ovipositor) which was measured from alcohol-preserved specimens before being dissected. All measurements are given in micrometres (μm) except body length which is measured in millimetres (mm). Scale bars are 100μm except those which are specifically indicated. All the specimens listed below are deposited in Northeast Forestry University, Harbin, China.

Terminology follows Hayat (1998) except mesosoma is used for the thorax plus propodeum and metasoma for the petiole plus gaster. The following abbreviations are used in the text:

F1−3: funicle segments 1−3

C1−3: clava segments 1−3

OAL: distance between a posterior ocellus and the anterior ocellus

OCL: distance between a posterior ocellus and occipital margin

OOL: distance between a posterior ocellus and eye margin

TI, TII, etc.: tergites 1, 2, etc. of gaster

The following acronyms are used for the depositories:

FACC: Fujian Agriculture and Forestry University, Fuzhou, Fujian, China

NEFU: Northeast Forestry University, Harbin, China

## Taxon treatments

### Pteroptrix pedicellata

Li & Chen, 2017 sp. n.

urn:lsid:zoobank.org:act:5F2936C8-208B-410A-BEAF-C9AE5B7BCFCC

#### Materials

- **Type status:** Holotype. **Occurrence:** recordedBy: Ye Chen; Chao Zhang; sex: female; lifeStage: adult; **Taxon:** scientificName: Pteroptrix pedicellataPteroptrix pedicellata; **Location:** country: China; stateProvince: Tibet; county: Chayu; municipality: Xiachayu Town; **Identification:** identifiedBy: Cheng-de Li; Ye Chen; dateIdentified: 2016-11; **Event:** samplingProtocol: sweeping; eventDate: 2015-05-15; **Record Level:** institutionCode: NEFU
- **Type status:** Paratype. **Occurrence:** recordedBy: Ye Chen; Chao Zhang; sex: 3 females; **Taxon:** scientificName: Pteroptrix pedicellataPteroptrix pedicellata; **Location:** country: China; stateProvince: Tibet; county: Chayu; municipality: Xiachayu Town; **Identification:** identifiedBy: Cheng-De Li; Ye Chen; dateIdentified: 2016-11; **Event:** samplingProtocol: sweeping; eventDate: 2015-05-15; **Record Level:** institutionCode: NEFU

#### Description

Female. Holotype. Body length 0.51 mm. Head dark brown, eyes black. Antenna mostly brown except pedicel and C1 paler. Thorax with pronotum, most part of mid-lobe of mesoscutum and axillae dark brown, side lobes and posterior part of mid-lobe of mesoscutum pale brown, scutellum and propodeum yellowish-white. Scutellum with light blue reflection in dorsal view. Wings hyaline, distinctly infuscated below marginal vein of fore wing, venation brown. Legs extensively dark brown, except fore coxae apically, mid coxae completely, apical half of all tibiae and tarsi pallid. Metasoma dark brown with the third valvula pale brown.

Head (Fig. 1a), in frontal view, about as long as wide; frontovertex about 0.5× head width. Ocellar triangle with apical angle obtuse. OOL larger than OCL and subequal to OAL. The sculpture and setation on the upper face as in a. Toruli with lower margins separated from mouth margin less than their own longest diameter and distance between toruli about as long as their longest diameter. Mandible tridentate. Antenna (Fig. 1b) with radicle 2.0× as long as wide, scape 4.75× as long as wide; pedicle 1.76× as long as wide and 1.20× as long as F1; F1 1.79× as long as wide, F2 subquadrate and 0.68× as long as F1; C1 2.3× as long as wide, a little longer than the two funicle segments combined; C2 about as long as or somewhat shorter than C1; C3 a little longer than both C1 and C2; three clava segments gradually decreasing in width distad. Measurements, length (width): radicle, 30 (15); scape, 95 (20); pedicle, 37.5 (21.3); F1, 31.3 (17.5); F2, 21.3 (20); clava 180 (25).

Mesosoma. Mid-lobe of mesoscutum and axillae with reticulate sculpture; scutellum with extremely faint reticulation. Mid-lobe of mesoscutum 0.73× as long as wide, with 12 nearly symmetrically arranged setae; each side lobe and axilla with 1 seta; scutellum 0.40× as long as wide and 0.63× as long as mid-lobe of mesoscutum. Placoid sensilla very close to the anterior scutellar setae; distance between anterior pair of scutellar setae 1.50× as long as the distance between posterior pair. Propodeum 2.60× the median length of metanotum and slightly salient posterior-medially. Mesopostphragma extending to posterior margin of TII. Fore wing (Fig. 1d) 3.55× as long as wide, densely and evenly setose; costal cell 1.58× as long as marginal vein, with 4 setae distally on dorsal surface and 4 relatively shorter setae medially on ventral surface; submarginal vein with 1 seta, marginal vein with 4 long setae along anterior margin; basal cell with 2 setae below apex of submarginal vein; marginal fringe 0.71× as long as the greatest width of disc. Hind wing 8.8× as long as wide, with 3 rows of setae on disc; marginal fringe 2.5× the greatest width of disc. Legs (Fig. 1e) with mid-tibial spur 1.31× as long as corresponding basitarsus. Measurements, length (width): fore wing, 495 (139.5); costal cell, 156.4; marginal vein, 99; stigmal vein, 30; marginal fringe, 99; hind wing, 435.6 (49.5); marginal fringe, 123.8; mid tibia, 158.4; mid-tibial spur, 45; mid basitarsus, 34.4; hind tibia, 178.2.

Metasoma (Fig. 1f). Ovipositor originating from base of TIV, 1.23× as long as mid-tibia and 1.09× as long as hind tibia, distinctly exserted at apex of gaster. Second valvifer 3.11× as long as third valvula, the latter 1.38× as long as mid basitarsus. Measurements: ovipositor, 195; second valvifer, 147.5; third valvula, 47.5.

Male. Unknown.

#### Diagnosis

Female. Body length 0.51−0.55mm. Body largely brown to dark brown; antenna (Fig. 1b) mostly brown except pedicel and C1 paler; legs (Fig. 1e) extensively dark brown, except mid coxae and apical half of all tibiae pallid. Antennal formula 1123, radicle 2.0× as long as wide, scape 4.56−4.75× as long as wide, about as long as the length of pedicel and funicle combined; pedicle 1.58−1.76× as long as wide and 1.11−1.31× as long as F1; F1 1.22−1.86× as long as wide; F2 subquadrate, 0.65−0.75× as long as F1; clava 3.27−3.60× as long as funicle. Funicle segments devoid of sensilla, each clava segment with 1 or 2 sensilla. Mid-lobe of mesoscutum (Fig. 1c) with 11−13 setae, each side lobe with 1 seta. Fore wing (Fig. 1d) 3.54−3.58× as long as wide and with its marginal fringe 0.71−0.77× wing width. Ovipositor 1.16−1.23× as long as mid-tibia and with its second valvifer 2.70−3.11× as long as third valvula. The third valvula 1.38−1.54× as long as mid basitarsus.

#### Host

Unknown.

#### Etymology

The specific name refers to the fact that this species is with normal sized pedicel in comparison with *Pteroptrix macropedicellata* (Malac).

#### Distribution

Tibet, Chayu County, Xiachayu Town

#### Comments

The new species belongs to the *dimidiata*-group (Viggiani and Garonna 1993) and superficially resembles *P. macropedicellata* (Malac) in having a similar body colour and setation on the mid-lobe of the mesoscutum. However, the new species can be separated from the latter (according to the description and figures of Nikol'skaya and Yasnosh (1966)) by the following combination of characters. *Pteroptrix pedicellata* sp. n. with relatively longer antennal scape, about as long as the combined length of pedicel, F1 and F2 (*vs* about as long as pedicel and F1 combined); normal sized pedicel, 1.58−1.76× as long as wide (*vs* 2×) and distinctly shorter than F1 + F2 (*vs* about as long as F1 + F2); shorter F1, 1.22−1.86× as long as wide (*vs* more than 2×); side lobe of mesoscutum with only 1 seta (*vs* 2 setae); relatively narrower fore wing, 3.54−3.58× as long as wide (*vs* about 3.11×).

### Pteroptrix leptocera

(Huang) 1992

Archenomus leptocerus Huang: *Huang et al. (1992)*: 167. Holotype female, China, Fujian, FACC, not examined.
Archenomus leptocerus : *Huang (1994)*: 120; Xu and Huang (2004): 311.
Pteroptrix leptocera : Viggiani and Ren (1993): 237.

#### Materials

- **Type status:** Other material. **Occurrence:** recordedBy: Xiang-xiang Jin; Guo-hao Zu; Chao Zhang; sex: 2 females; lifeStage: adult; **Taxon:** scientificName: Pteroptrix leptoceraPteroptrix leptocera; originalNameUsage: *Archenomus leptocerusArchenomus leptocerus* Huang, 1992; **Location:** country: China; stateProvince: Yunnan; county: Lianghe; **Identification:** identifiedBy: Cheng-De Li; Ye Chen; dateIdentified: 2016-11; **Event:** samplingProtocol: yellowpan trapping; eventDate: 2013 04-29/05-01; **Record Level:** institutionCode: NEFU

#### Host

Unknown

#### Distribution

China (Fujian, Yunnan [**new record**]).

#### Comments

Although this species was adequately described and illustrated by Huang et al. (1992), this is the first record from Yunnan Province, China.

### Pteroptrix orientalis

(Silvestri) 1909

Archenomus orientalis Silvestri 1909: 563. Italy, not examined.
Archenomus orientalis : Prinsloo and Neser (1990): 24; Huang et al. (1992): 164; Huang (1994): 103; Xu and Huang (2004): 313.
Pteroptrix orientalis : Viggiani and Garonna (1993): 59; Viggiani and Ren (1986): 237.

#### Materials

- **Type status:** Other material. **Occurrence:** recordedBy: Cheng-De Li; sex: 1 female; lifeStage: adult; **Taxon:** scientificName: Pteroptrix orientalisPteroptrix orientalis; originalNameUsage: *Archenomus orientalisArchenomus orientalis* Silvestri 1909; **Location:** country: China; stateProvince: Liaoning; county: Dalian City; municipality: Pulandian; **Identification:** identifiedBy: Cheng-De Li; **Event:** samplingProtocol: sweeping; eventDate: 1994-06-05; **Record Level:** institutionCode: NEFU
- **Type status:** Other material. **Occurrence:** recordedBy: Xiang-xiang Jin;Guo-hao Zu;Si-zhu Liu; sex: 3 females; lifeStage: adult; **Taxon:** scientificName: Pteroptrix orientalisPteroptrix orientalis; **Location:** country: China; stateProvince: Shandong; county: Qingdao City; municipality: Xiaozhu Mountain; **Identification:** identifiedBy: Cheng-De Li; Ye Chen; dateIdentified: 2016-12; **Event:** samplingProtocol: sweeping; eventDate: 2014-5-20; **Record Level:** institutionID: NEFU

#### Host

*Pseudaulacaspis pentagona* (Targioni-Tozzetti) (Hemiptera:Diaspididae). (Huang (1994)).

#### Distribution

China (Liaoning [**new record**], Shandong [**new record**], Fujian), South Korea, Japan, Italy, Dominican Republic (Noyes 2016).

#### Comments

**Figure 2.**
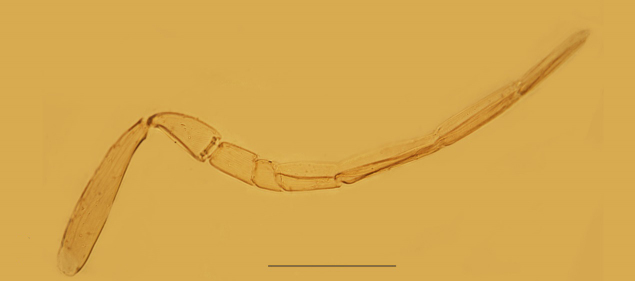
*Pteroptrix orientalis*, female: Antenna.

Our specimens agree well with the description of Huang (1994), but the following difference should be noted: in the material from Qingdao City, F3 as in Fig. 2 with one sensillum (sensilla present only on clava segments in Huang^'^s description).

### Pteroptrix processa

(Huang) 1991

Archenomus processus Huang (1991): 396. Holotype female, China, Jiangsu, FACC, not examined.
Archenomus processus : Huang (1994): 114; Xu and Huang (2004): 316.
Pteroptrix processa : Viggiani and Ren (1993): 237; Viggiani and Garonna (1993): 61.

#### Materials

- **Type status:** Other material. **Occurrence:** recordedBy: Xiu-Wei Liu; sex: 1 female; lifeStage: adult; **Taxon:** scientificName: Pteroptrix processaPteroptrix processa; originalNameUsage: *Archenomus processusArchenomus processus* Huang 1991; **Location:** country: China; stateProvince: Beijing City; municipality: Chinese Academy of Sciences, Institute of Botany; **Identification:** identifiedBy: Cheng-De Li; Ye Chen; dateIdentified: 2016-11; **Event:** samplingProtocol: yellowpan trapping; eventDate: 2012-05-09; **Record Level:** institutionID: NEFU
- **Type status:** Other material. **Occurrence:** recordedBy: Ye Chen; Chao Zhang; sex: 1 male; lifeStage: adult; **Taxon:** scientificName: Pteroptrix processaPteroptrix processa; **Location:** country: China; stateProvince: Shaanxi; county: Ningshan; municipality: Guanghuojie Town; **Identification:** identifiedBy: Cheng-De Li; Ye Chen; dateIdentified: 2016-1; **Event:** samplingProtocol: sweeping; **Record Level:** institutionID: NEFU

#### Description

Male. Body length 0.5 mm. Colouration similar to that of female, largely brown to dark brown. Antenna (Fig. 3a) with radicle 2.73× as long as wide; scape 4.33× as long as wide; pedicle slightly longer than wide and 0.63× as long as F1; funicle segments with F2 shortest, F3 a little longer than F1. Mid-lobe of mesoscutum with 2 setae, propodeum distinctly salient posterior-medially and 3.6 × as long as metanotum. Fore wing (Fig. 3b) 2.8 × as long as wide, marginal fringe 0.48 × as long as the greatest width of wing. Genitalia as in Fig. 3c and 0.55 × as long as midtibia.

#### Host

*Pseudaulacaspis pentagona* (Targioni-Tozzetti) (Hemiptera: Diaspididae), (Huang 1991).

#### Distribution

China (Fujian, Jiangsu, Beijing [**new record**], Shaanxi [**new record**]).

#### Comments

This species is unique for the genus in having the mid-lobe of mesoscutum with only 2 setae and propodeum distinctly salient posterior-medially. This is the first report for its male.

## Supplementary Material

XML Treatment for Pteroptrix pedicellata

XML Treatment for Pteroptrix leptocera

XML Treatment for Pteroptrix orientalis

XML Treatment for Pteroptrix processa

## Figures and Tables

**Figure 1a.**
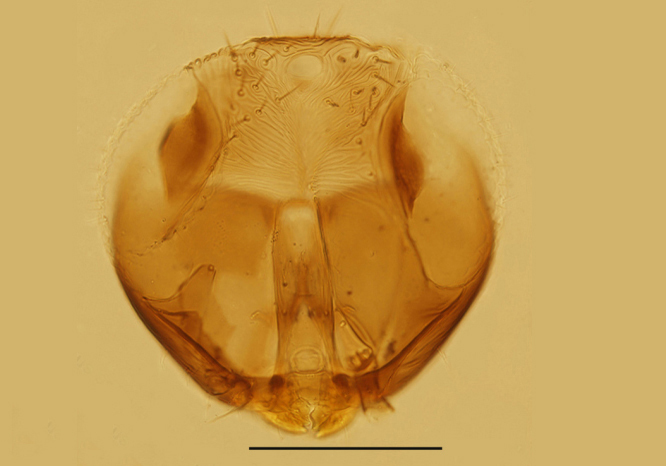
Head, frontal view

**Figure 1b.**
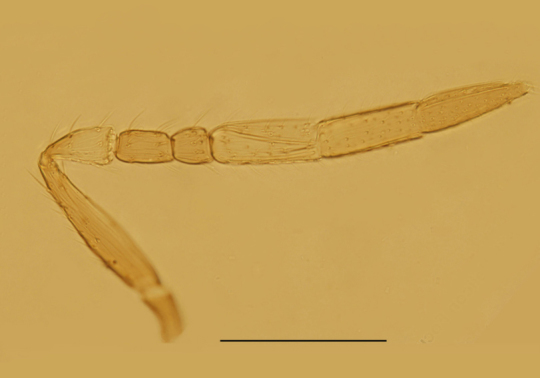
Antenna

**Figure 1c.**
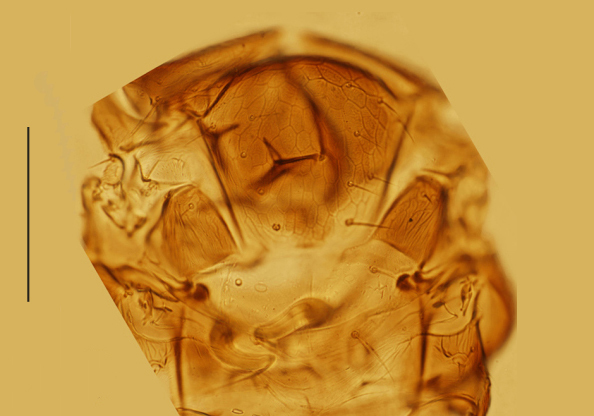
Mesosoma, dorsal from paratype

**Figure 1d.**
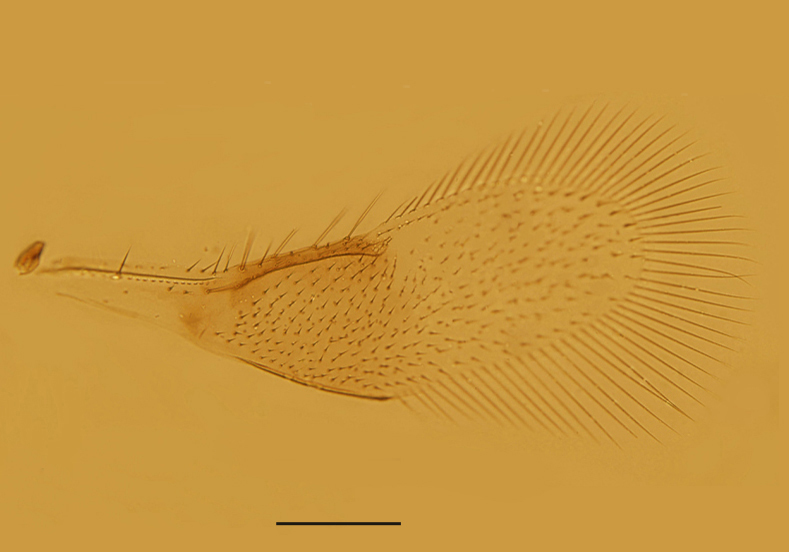
Fore wing

**Figure 1e.**
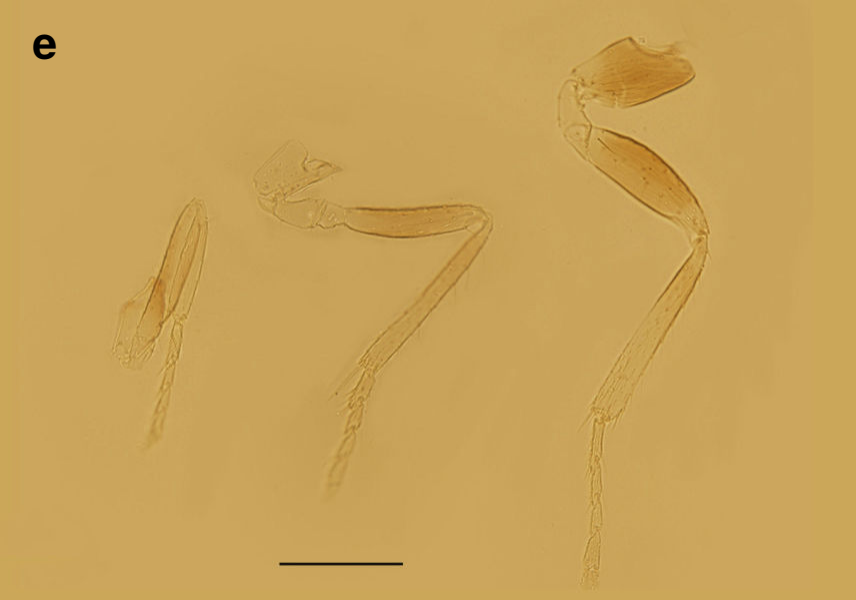
Legs

**Figure 1f.**
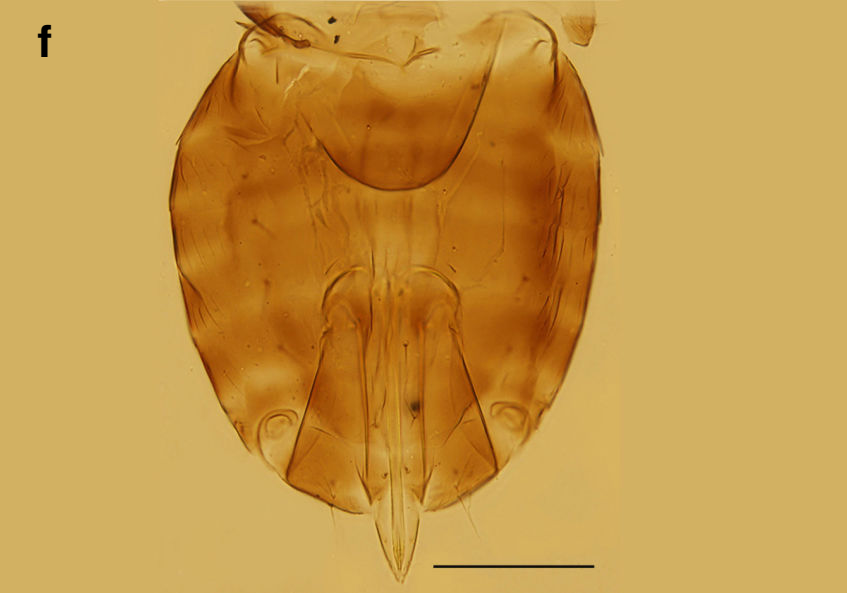
Metasoma, dorsal

**Figure 3a.**
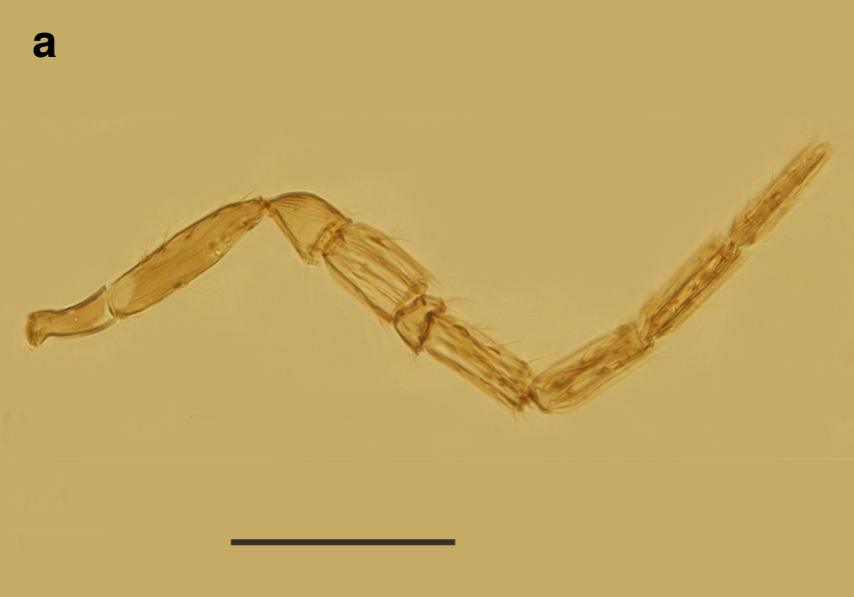
Antenna

**Figure 3b.**
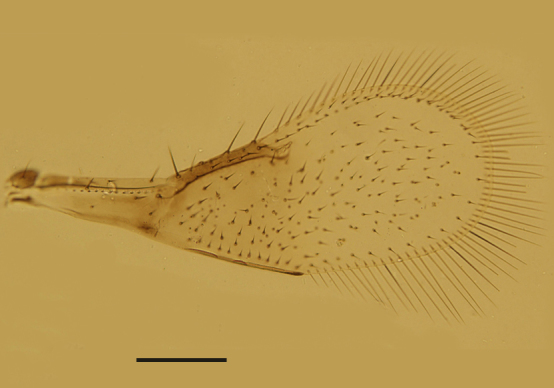
Fore wing

**Figure 3c.**
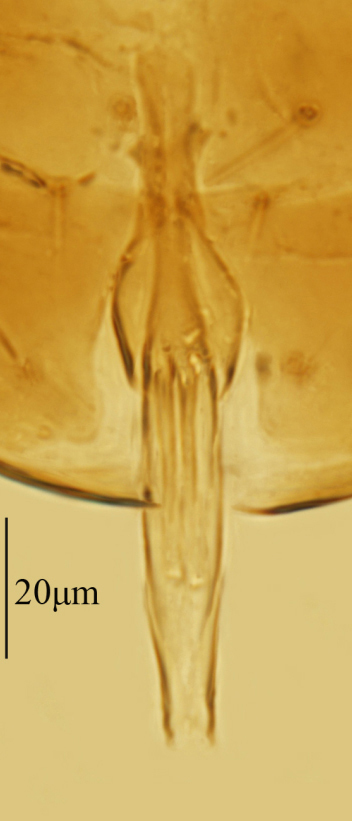
Genitalia

## References

[B3571916] Abd-Rabou S. (2002). Revision of Aphelinidae (Hymenoptera) from Egypt.. Second International Conference of Plant Protection Research Institute, Dokki, Giza, Egypt.

[B3571926] Compere H. (1953). An appraisal of Silvestri's work in the Orient for the University of California, some misidentifications corrected and two forms of *Casca* described as new species.. Bollettino del Laboratorio di Zoologia Generale e Agraria della Facoltà Agraria in Portici.

[B3572126] Flanders S. E. (1966). Unique biological aspects of the genus *Casca* and a description of a new species (Hym. Aphelinidae).. Annals of the Entomological Society of America.

[B3572136] Hayat M (1979). The tetramerous Aphelinidae (Hymenoptera) of Girault from Australia, with a key to the World genera.. Systematic Entomology.

[B3572146] Hayat M (1998). Aphelinidae of India (Hymenoptera: Chalcidoidae): A taxonomic revision.. Memoirs on Entomology.

[B3572156] Hayat M, Khan F. R. (2010). Additions to the Aphelindae of India (Hymenoptera: Chalcidoidea): 1. On species of *Ablerus* Howard, *Coccobius* Ratzeburg, *Coccophagus* Westwood, *Pteroptrix* Westwood, and *Idiococcobius* Hayat gen. nov.. Colemania.

[B3572166] Howard L. O. (1907). New genera and species of Aphelininae with a revised table of genera.. Technical Series, Bureau of Entomology, United States Department of Agriculture.

[B3572191] Huang J (1991). Systematic studies of Aphelinidae II. Descriptions of four new species of *Archenomus* Howard from China (Hymenoptera: Aphelinidae).. Journal of Fujian Agricultural College.

[B3572201] Huang J (1994). Systematic studies on Aphelinidae of China (Hymenoptera: Chalcidoidea)..

[B3572210] Huang J, Lin X. L., Lin C. F. (1992). Systematic studies of Aphelinidae III. The species of *Archenomus* Howard from China (Hymenoptera: Aphelinidae).. Journal of Fujian Agricultural College.

[B3572220] Myartseva S. N. (2009). Two new species of *Pteroptrix* Westwood, 1833 (Hymenoptera: Aphelinidae) from Mexico.. Zoosystematica Rossica.

[B3572230] Nikol'skaya M. N., Yasnosh V. A. (1966). Aphelinids of the European part of the USSR and the Caucasus (Hymenoptera: Aphelinidae). Opredeliteli po Faune SSSR.

[B3572240] Noyes J. S. (1982). Collecting and preserving chalcid wasps (Hymenoptera: Chalcidoidea).. Journal of Natural History.

[B3572250] Noyes J. S. Universal Chalcidoidea Database. World Wide Web electronic publication.. http://www.nhm.ac.uk/chalcidoids.

[B3572259] Prinsloo G. L., Neser O. C. (1990). The southern African species of *Archenomus* Howard (Hymenoptera: Aphelinidae) with a key to species of the World.. Entomology Memoir of the Department of Agricultural Development of the Republic of South Africa.

[B3572269] Silvestri F. (1909). Notizie e descrizioni preliminari di insetti parassiti della *Diaspispentagona*.. Attidella Reale Accademia dei Lincei. Rendiconti. Roma. Classe Scienze Fiziche, Matematiche e Naturali.

[B3572289] Viggiani G., Garonna A. P. (1993). Le specie italiane del complesso *Archenomus* Howard, *Archenomiscus* Nikol'skaja, *Hispaniella* Mercet e *Pteroptrix* Westwood, con nuove combinazioni generiche (Hymenoptera: Aphelinidae).. Bollettino del Laboratorio di Entomologia Agraria 'Filippo Silvestri'.

[B3572279] Viggiani G., Ren H. (1986). Two new aphelinids from China (Hymenoptera: Chalcidoidea).. Bollettino del Laboratorio di Entomologia Agraria 'Filippo Silvestri'.

[B3572299] Viggiani G., Ren H. (1993). New species and records of Aphelinidae (Hymenoptera: Chalcidoidea) from China.. Bollettino del Laboratorio di Entomologia Agraria 'Filippo Silvestri.

[B3572602] Westwood J. O. (1833). Descriptions of several new British forms amongst the parasitic hymenopterous insects. Philosophical Magazine (3).

[B3572319] Xu Z. H., Huang J. (2004). Chinese fauna of parasitic wasps on scale insects..

